# Preoperative Functional Disability and Perioperative Life-space Mobility in Older Patients with Lumbar Spinal Stenosis: A Generalized Linear Mixed-effects Model Analysis

**DOI:** 10.1298/ptr.25-E10369

**Published:** 2026-02-10

**Authors:** Daigo ISHIZUKA, Susumu NOZAKI, Hiroshi MINEZAKI, Tsuyoshi OTA, Yasuyoshi ASAKAWA

**Affiliations:** 1 Department of Physical Therapy, Graduate School of Human Health Sciences, Tokyo Metropolitan University, Japan; 2 Department of Physical Therapy, Faculty of Rehabilitation Science, Faculty of Health Sciences, University of Human Sciences, Japan; 3 Nissay Seirei Health and Welfare Foundation, Matsudo Nissay Seirei Clinic, Japan; 4 Department of Rehabilitation Medicine, Saitama Saiseikai Kawaguchi General Hospital, Saitama Branch of Saiseikai Foundation, Japan; 5 Department of Physical Therapy, Department of Human Health Sciences, Tokyo Metropolitan University, Japan

**Keywords:** Lumbar spinal stenosis, Oswestry Disability Index, Life-Space Assessment, Generalized linear mixed-effects model, Older adult

## Abstract

**Objectives:**

This prospective cohort study investigated the relationship between preoperative functional disability, as determined by the Oswestry Disability Index (ODI), and perioperative Life-Space Assessment (LSA) scores using generalized linear mixed-effects models (GLMM).

**Methods:**

We included 262 patients (mean age, 76.1 ± 6.0 years; male, n = 140; female, n = 122) with lumbar spinal stenosis scheduled for surgery. LSA was examined preoperatively and at 3 and 6 months postoperatively, while ODI was assessed preoperatively. Patients were classified based on ODI quartiles (Q1: 0%–28.89%, Q2: 28.89%–40.0%, Q3: 40.0%–53.33%, and Q4: 53.33%–86.67%). We explored the relationship between ODI and LSA using GLMM.

**Results:**

The mean preoperative ODI score was 41.2% ± 17.1%. In the crude model, ODI showed a significant negative association with LSA (β = −0.38, 95% CI: −0.58 to −0.18); this relationship remained significant in the adjusted model (β = −0.30, 95% CI: −0.49 to −0.11). Older age (β = −1.20, 95% CI: −1.76 to −0.65) and female sex (β = −11.81, 95% CI: −18.24 to −5.38) were associated with life-space restriction; body mass index, number of decompressed levels, and comorbidity burden did not show any such significant associations.

**Conclusions:**

Preoperative functional disability significantly affected perioperative life-space mobility among older patients with lumbar spinal stenosis. A comprehensive assessment from the preoperative period is important for older female patients.

## Introduction

Lumbar spinal stenosis (LSS) is a degenerative disease characterized by spinal canal narrowing and nerve compression that frequently affects older patients^1,2)^. The prevalence of LSS and the number of spinal surgeries are increasing in aging societies^2–4)^. Patients with LSS often experience lower limb pain, walking difficulties, and a decreased health-related quality of life (QOL), which are associated with a decline in activities of daily living (ADL)^5–7)^. When conservative treatment does not sufficiently improve LSS symptoms, they can be surgically relieved^8)^, but its effects vary among patients. Postoperative symptoms can persist, especially in patients with severe preoperative symptoms^4,9)^.

A decline in ADL caused by functional disability is thought to lead to life-space constriction among older persons^10,11)^. Life-space reflects not only mobility distance but also social participation and independence, and its restrictions are associated with social isolation and mortality^11,12)^. Thus, patients with LSS and functional disability due to its symptoms might have restricted life-spaces. Understanding the actual status of ADL and life-spaces is important for managing and treating older patients with LSS. However, longitudinal studies of the relationship between preoperative functional disability and perioperative life-space mobility are limited. Clarifying this relationship is important for comprehensively understanding postoperative outcomes among older patients with LSS and developing appropriate intervention strategies^13,14)^.

The aim of this study was to determine the relationship between preoperative functional disability and perioperative life-space in older patients with LSS using generalized linear mixed-effects models (GLMM). We tested the hypothesis that preoperative functional impairment affects postoperative life-space in older patients with LSS.

## Methods

### Study design and participants

This prospective cohort study consecutively recruited patients diagnosed with LSS who were surgically treated at a dedicated spinal unit at a single institution. The inclusion criteria were: LSS diagnosed by a spine specialist, age ≥65 years, and scheduled for surgery. The exclusion criteria were walking disabilities due to lower limb fractures, hemiplegia, neuromuscular diseases, inability to respond to questionnaires due to cognitive impairment, and restricted ADL due to heart failure or other diseases.

Patients were admitted 1 day before surgery, and physical therapy commenced on the day after surgery. The average hospital stay was approximately 2 weeks. The physical therapy program consisted primarily of exercise therapy for functional impairment, with the goal of achieving independent walking within 1 week postoperatively. In addition, patients received instructions on prohibited lumbar spine movements and guidance on post-discharge activities and social participation. Following discharge, no outpatient physiotherapy sessions were conducted at the affiliated facility. Data were collected from 262 patients who met the inclusion criteria and provided consent among 413 patients with LSS scheduled for surgery between October 1, 2021 and March 31, 2024. Questionnaire surveys were conducted during outpatient visits. The Institutional Ethics Committee at Tokyo Metropolitan University and Saiseikai Kawaguchi General Hospital approved this study (Approval ID: 21050, 2021-20), which complied with the ethical principles enshrined in the Declaration of Helsinki (2013 amendment). Written informed consent was obtained from all participating patients. We included all patients who could be registered during the study period.

### Evaluated items

#### Basic information

We collected data from the electronic medical records. Data on patient characteristics included age, sex, body mass index (BMI), comorbidity, and surgical information. Comorbidity was assessed using the Functional Comorbidity Index (FCI)^15)^, which includes conditions specifically associated with physical function. Each condition was assigned 1 point (range: 0–18), with higher scores indicating greater comorbidity burden. Based on the FCI categories, the most common comorbidities affecting physical function were visual impairment, including cataracts and glaucoma (29.1%), degenerative disc disease (24.6%), and diabetes mellitus (18.9%). Surgical procedures were classified into decompression with fusion (58.6%) and decompression only (41.4%). The operated segments were categorized as single-level (38.9%) or multi-level (61.1%).

#### Life-Space Assessment

We evaluated the frequency and extent to which older community dwellers went outside the home using the Life-Space Assessment (LSA) questionnaire^16,17)^ preoperatively, at 3 months postoperatively, and at 6 months postoperatively. The LSA has established validity (convergent validity with functional measures and content validity) and reliability (Cronbach’s α = 0.80–0.92; intraclass correlation coefficient [ICC] = 0.89–0.97)^16,17)^.

We calculated LSA scores based on the range of activities, their frequency, and the degree of independence experienced during the past month. The activity range was categorized as inside and outside the home, in the neighborhood (within 800 m) and town (>800 m and ≤16 km), and outside the town (>16 km). The final calculated score was the sum of scores for each activity range (range: 0–120), with higher values indicating more life-space and a higher frequency of activity^17)^.

#### Functional disabilities associated with lower back pain

We evaluated functional disabilities associated with lower back pain using the Oswestry Disability Index (ODI) questionnaire (validity: construct validity established with correlations to functional measures; reliability: Cronbach’s α = 0.80–0.90, test–retest reliability ICC >0.90)^18,19)^, which collects data by asking about pain intensity, personal care, lifting, walking, sitting, standing, sleeping, sex life, social life, and travel. The sections were all scored from 0 to 5 and are expressed as ratios (%) of the maximum score. Unanswered sections were resolved by dividing the accumulated score by 50 minus the number of answered sections multiplied by 5, resulting in a range of 0–100. We evaluated 9 items, excluding questions about sex life, in consideration of national cultural practices. Higher scores indicate more severe functional disability^18)^.

#### Statistical analysis

We classified patients into 4 groups based on ODI quartiles (Q1: 0%–28.89%, Q2: 28.89%–40.0%, Q3: 40.0%–53.33%, and Q4: 53.33%–86.67%) to evaluate baseline characteristics. We performed Kruskal–Wallis tests for continuous variables, followed by Bonferroni post hoc tests when significant differences were found. For categorical variables, we performed χ^2^ tests and residual analysis. We analyzed the relationship between ODI and LSA using GLMM to construct 2 models for the primary analysis:

1. Crude model: LSA = ODI + time + (1|ID)
2. Adjusted model: LSA = ODI + age + sex + BMI + segments + FCI + time + (1|ID)

Segments were used to classify into single- and multi-level categories. Measurements were taken at 3 time points: preoperatively (baseline), 3 months postoperatively, and 6 months postoperatively. We included GLMM because its hierarchical structure accommodates repeated measurement data from individual patients and incorporates random effects to account for inter-individual heterogeneity. To evaluate model explanatory power, we calculated marginal R^2^ (variance explained by fixed effects alone) and conditional R^2^ (variance explained by the entire model, including both fixed and random effects)^20)^. Follow-up rates and potential bias due to loss to follow-up were evaluated using a sensitivity analysis. Baseline characteristics were compared between patients who had been preoperatively evaluated and had completed 6 months of postoperative follow-up and those who were lost to follow-up. Continuous variables and categorical variables were compared using Mann–Whitney U tests and chi-squared tests, respectively.

All data were statistically analyzed using R version 4.4.0 (R Foundation for Statistical Computing, Vienna, Austria). Values with p <0.05 were considered statistically significant.

## Results

### Characteristics of participants

**Fig. 1.**
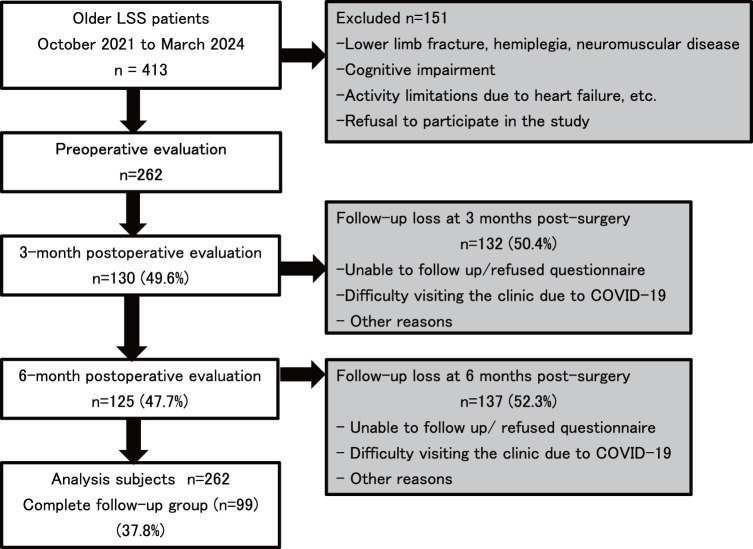
Flowchart of patients through the study. LSS, lumbar spinal stenosis; COVID-19, coronavirus disease 2019

The study included 262 patients (male, n = 140; female, n = 122; mean age, 76.1 ± 6.0 years), 99 (37.8%) of whom completed follow-up (Fig. 1). The mean preoperative ODI was 41.2% ± 17.1%. Among the 4 ODI quartile groups, age and LSA significantly differed after Bonferroni correction (Table 1). The Q4 group (highest ODI) was significantly older and had lower preoperative LSA scores than the other groups.

**Table 1.** Baseline characteristics of study participants

	Total (n = 262)	Q1 (n = 57)	Q2 (n = 72)	Q3 (n = 69)	Q4 (n = 64)	p
Age (years)	76.1 ± 6.0	74.9 ± 5.6	76.1 ± 5.9	75.1 ± 5.5	78.3 ± 6.5^a,b,c^	0.006*
Sex						0.145
Male	140 (53.4)	33 (57.9)	36 (50.0)	43 (62.3)	28 (43.8)	
Female	122 (46.6)	24 (42.1)	36 (50.0)	26 (37.7)	36 (56.3)	
BMI (kg/m^2^)	24.0 ± 3.6	23.5 ± 3.2	24.1 ± 3.8	24.1 ± 3.5	24.2 ± 3.8	0.876
FCI score	1.4 ± 1.1	1.5 ± 1.1	1.5 ± 1.0	1.6 ± 1.0	1.3 ± 1.3	0.523
Surgical procedure						0.996
Fixation	154 (58.8)	33 (57.9)	42 (58.3)	41 (59.4)	38 (59.4)	
Decompression	108 (41.2)	24 (42.1)	30 (41.7)	28 (40.6)	26 (40.6)	
Segments						0.058
Single-level	105 (40.1)	26 (45.6)	19 (26.4)	31 (44.9)	29 (45.3)	
Multi-level	157 (59.9)	31 (54.4)	53 (73.6)	38 (55.1)	35 (54.7)	
Preoperative ODI (%)	41.2 ± 17.1	20.1 ± 8.5	35.7 ± 3.0^f^	47.9 ± 3.6^d,e^	63.7 ± 8.4^a,b,c^	<0.001*
Median (IQR)	40.0 (28.9–53.3)	20.0 (11.1–26.7)	35.6 (33.3–37.8)	47.8 (44.4–51.1)	62.2 (57.8–68.9)	
Follow-up period, n (%)						
Preoperative	262 (100)	57 (21.8)	72 (27.5)	69 (26.3)	64 (24.4)	
3 months postoperative	130 (49.6)	31 (54.4)	36 (50.0)	35 (50.7)	28 (43.8)	
6 months postoperative	125 (47.7)	26 (45.6)	36 (50.0)	32 (46.4)	31 (48.4)	
LSA						
Preoperative	60.2 ± 33.5	66.9 ± 30.4	64.3 ± 33.9	61.0 ± 33.8	48.7 ± 33.3^a^	0.012*
3 months postoperative	72.5 ± 30.6	78.3 ± 26.4	71.4 ± 29.7	70.2 ± 32.1	67.6 ± 38.4	0.456
6 months postoperative	82.5 ± 30.6	87.4 ± 24.3	84.6 ± 29.8	81.8 ± 31.2	72.5 ± 37.5	0.098

*p <0.05.

^a^ Q1–Q4.

^b^ Q2–Q4.

^c^ Q3–Q4.

^d^ Q1–Q3.

^e^ Q2–Q3.

^f^ Q1–Q2.

BMI, body mass index; FCI, Functional Comorbidity Index; ODI, Oswestry Disability Index; IQR, interquartile range; LSA, Life-Space Assessment

### Relationship between ODI and LSA

In the crude model, ODI showed a negative association with LSA (β = −0.38, 95% CI: −0.58 to −0.18) (Table 2), with a marginal R^2^ of 0.152 and conditional R^2^ of 0.361. After adjusting for age, sex, BMI, segments and FCI, the association remained significant (β = −0.28, 95% CI: −0.47 to −0.09); older age (β = −1.20, 95% CI: −1.76 to −0.65) and female sex (β = −11.81, 95% CI: −18.24 to −5.38) also showed negative associations with LSA (Table 3). In the adjusted model, the marginal R^2^ was 0.189 and the conditional R^2^ was 0.523, indicating that fixed effects explained approximately 19% of the variance, while the entire model explained approximately 52% of the variance. LSA significantly improved at 3 months (β = 10.21, 95% CI: 4.81–15.60) and 6 months (β = 21.97, 95% CI: 15.34–28.60) postoperatively compared with the preoperative values. The comparison of baseline characteristics revealed a significantly lower preoperative ODI among the patients who completed the follow-up than among those who did not (39.4% ± 17.1% vs. 44.1% ± 16.1%, p = 0.025) (Table 4).

**Table 2.** Crude model: Association between ODI and LSA

Variable	β	SE	95% CI	p
Intercept	76.36	4.67	(67.20 to 85.52)	<0.001
ODI (per 1% increase)	−0.38	0.10	(−0.58 to −0.18)	<0.001
3 months post- vs. preoperative	10.04	2.78	(4.59 to 15.49)	<0.001
6 months post- vs. preoperative	20.45	2.81	(14.94 to 25.96)	<0.001

Model fit indices: AIC, 4835.8; BIC, 4856.4; conditional R^2^, 0.361; marginal R^2^, 0.152.

ODI, Oswestry Disability Index; LSA, Life-Space Assessment; SE, standard error; CI, confidence interval; AIC, Akaike Information Criterion; BIC, Bayesian Information Criterion

**Table 3.** Adjusted model: Association between ODI and LSA

Variable	β	SE	95% CI	p
Intercept	185.30	25.96	(134.41 to 236.19)	<0.001
ODI (per 1% increase)	−0.28	0.10	(−0.47 to −0.09)	0.004
Age (per 1-year increase)	−1.20	0.28	(−1.76 to −0.65)	<0.001
Sex (female vs. male)	−11.81	3.28	(−18.24 to −5.38)	<0.001
BMI (per 1 increase)	−0.71	0.45	(−1.60 to 0.18)	0.121
Segments (single/multi)	0.73	3.35	(−5.84 to 7.31)	0.828
FCI (per 1 increase)	0.29	1.45	(−2.55 to 3.13)	0.842
3 months post- vs. preoperative	10.21	2.75	(4.81 to 15.60)	<0.001
6 months post- vs. preoperative	21.97	3.38	(15.34 to 28.60)	<0.001

Model fit: AIC 4829.9, BIC 4876.3, conditional R^2^ 0.523, marginal R^2^ 0.189.

ODI, Oswestry Disability Index; LSA, Life-Space Assessment; SE, standard error; CI, confidence interval; BMI, body mass index (kg/m^2^); FCI, Functional Comorbidity Index; AIC, Akaike Information Criterion; BIC, Bayesian Information Criterion

**Table 4.** Comparison of baseline characteristics between patients who completed the study and those who were lost to follow-up

Characteristic	Completed (n = 99)	Lost (n = 163)	p
Age (years)	75.7 ± 6.0	76.4 ± 6.0	0.340
Sex (male/female)	48/51	92/712	0.105
BMI (kg/m^2^)	24.1 ± 4.2	23.9 ± 3.3	0.736
Segments	1.4 ± 1.1	1.5 ± 1.1	0.793
FCI	34/65	66/97	0.710
Preoperative ODI (%)	39.4 ± 17.1	44.1 ± 16.1	0.025*
Preoperative LSA	62.7 ± 31.9	58.7 ± 34.2	0.342

*p <0.05.

Values are shown as means ± standard deviation or frequency.

BMI, body mass index; FCI, Functional Comorbidity Index; ODI, Oswestry Disability Index; LSA, Life-Space Assessment

## Discussion

This study examined the association between preoperative functional disability and perioperative life-space among older patients with LSS using GLMM. Among the 262 patients analyzed, higher preoperative ODI scores were significantly associated with lower perioperative LSA scores, with this relationship remaining consistent across the study period.

Scores for LSA differing by 5–10 and 15 are clinically meaningful^21,22)^. This indicates that the perioperative life-space differs among patients. The preoperative ODI score associated with life-space suggests that functional disability affects mobility, probably due to intermittent claudication and psychological factors^23,24)^. Distances walked decrease as intermittent claudication worsens^23)^; this affects participation in social activities^25)^. Therefore, patients might be forced to gradually limit activities to progressively narrower living areas. Improvements in LSA were significant 3 and 6 months postoperatively compared with preoperative values. Our findings of evident functional recovery 3 and 6 months after surgery for LSS^26,27)^ were consistent with the life-space results. The improvement in LSA over the postoperative course suggests that patients gradually expand their activity range in line with functional recovery, with consistent improvement patterns observed across different levels of preoperative functional disability.

We clarified the independent effects of age and sex on life-space among patients aged ≥65 years with LSS. The adjusted model showed that older age and female sex were significantly associated with life-space restriction. Degenerative changes cause LSS, the incidence of which increases with aging^1)^. The comparison between crude and adjusted models revealed that the association between ODI and LSA remained statistically significant even after controlling for age, sex, BMI, segments, and FCI. This consistency suggests that the relationship between functional disability and life-space mobility is not merely confounded by demographic factors. Decreased activity easily leads to a decline in physical function and limited ADL among older individuals^28,29)^. Furthermore, older individuals with limited ADL and restricted life-spaces not only have symptoms of LSS but also of complex factors associated with aging. Against this background, we specifically targeted patients aged ≥65 years with LSS and aimed to comprehensively capture complex problems specific to this population by combining LSA with conventional functional evaluation. Although previous preoperative findings have shown no significant differences in life-space between males and females^7)^, the present findings revealed that females had significantly more life-space restrictions than males for up to 6 months postoperatively. The postoperative outcomes of LSS, such as pain, functional disability, and health-related QOL, are lower in females than in males^30)^. However, the mechanisms underlying these sex-related differences remain unclear and require further investigation. These factors are considered to have a stronger postoperative effect on life-space constriction among females. Our results indicate the importance of individualized treatment approaches, considering age and sex in older patients with LSS up to 6 months postoperatively. Specifically, if the preoperative ODI severity is high, the patient will not be able to obtain the life-space that they would normally be able to. If life-space expansion of the patient is possible, intensive rehabilitation intervention is recommended. If this is difficult, consideration should be given to assistive devices, environmental adjustments, and early surgery. Although sex cannot be adjusted, surgery is recommended before the ODI worsens or the patient reaches advanced age.

This study has some important limitations. Overall, 62.2% of data were lost to follow-up owing to attrition, which is common in longitudinal studies of older individuals^31)^. However, baseline characteristics, except for preoperative ODI scores, did not significantly differ between groups. Because the follow-up group included patients with relatively mild LSS, our findings might have overestimated the overall postoperative course. Furthermore, this single-institution observational study limits external validity. The conditional R^2^ (0.523) indicates considerable within-subject variability remains unexplained, suggesting that unmeasured factors such as psychological factors (e.g., depression, anxiety, fear of movement), social support, economic status, and living environment play important roles. Moreover, we did not systematically record detailed rehabilitation protocols or assess preoperative lower-extremity function and physical capacity, limiting our ability to interpret the observed associations. Future studies should include the detailed documentation of postoperative interventions and preoperative physical function assessments.

We confirmed that evaluating the preoperative ODI is essential to identify patients who need individualized intervention strategies. Our findings suggest that preoperative ODI assessment can guide individualized rehabilitation strategies. Specifically, patients with high preoperative ODI scores, particularly older females, may benefit from enhanced preoperative rehabilitation interventions as well as strengthened post-discharge life support, including social reintegration support.

## Conclusions

Patients with higher preoperative ODI had lower perioperative LSA scores, and older females were associated with life-space restriction. In the adjusted model, BMI, operated segments, and comorbidity burden did not significantly affect LSA. Comprehensive intervention from the preoperative period is essential for older females and patients with preoperative functional disability.
